# Books

**Published:** 1883-04

**Authors:** 


### Books.

A PRACTICAL TREATISE ON DISEASES OF THE SKIN, FOR THE USE OF STUDENTS AND PRACTITIONERS. By James Nevins Hyde, A.M., M.D. Philadelphia: Henry C. Lea's Son & Co. 1883. Pp. 572.

This handsome volume has been prepared by the author for the convenience of the general practitioner, and it has been his aim to present herein, in a comprehensive form, the results of the latest observation and experience in this important and often perplexing department of medicine.

After a careful examination of the work, we can truthfully say that the author has discharged the task set before him with a fidelity and completeness that reflects great credit upon him, and in such a manner as, we doubt not, will place the treatise among the very best and most trustworthy of all those relating to this subject.

Our space will not admit of an extended review, but we can conscientiously commend the book to the medical profession as a reliable and valuable exponent of the latest and most reliable doctrines extant pertaining to the science of dermatology.

---

Experimental Pharmacology. A Hand-book of Methods for Studying the Physiological Actions of Drugs. By L. Hermann, Professor of Physiology at the University of Zurich. Translated, with the author's permission, with notes and additions. By Robert Meade Smith, M.D.; with thirty-two illustrations on wood. Philadelphia: Henry C. Lea's Son & Co. 1883. Pp. 201.

The additions of the translator, with the illustrations which he has introduced, constitute nearly one-half of this little volume, and he explains his object in translating this manual to be a desire to assist the student in studying the physiological action of drugs.

Part first is dedicated to the study of the action of poisons on isolated organs—the blood, muscles, nerves and heart. Part second comprises investigations into the general actions of poisons. The character of the work is sufficiently defined in the title and by the above reference to its contents. It will prove a serviceable guide and assistant to those engaged in studies and investigations of this kind.

---

The Systematic Treatment of Nerve Prostration and Hysteria. By W. S. Playfair, M.D., F.R.C.S. Philadelphia: Henry C. Lea's Son & Co. 1883. Pp. 111.

This little book represents the views of the author as to the treatment of certain troublesome nervous disorders of the hysterical and neurasthenic type. The system of treatment herein advocated was suggested to the author through the study of a little work by Dr. Weir Mitchell, of Philadelphia, entitled "Fat and Blood, and How to Make Them," which was published several years ago.

"Although," says the author, "Dr. Mitchell's treatment was not new in the sense that its separate recommendations were made for the first time, it was new in the sense that these recommendations were, for the first time, combined so as to form a complete scheme of treatment."

Massage is regarded as a very important part of the treatment in certain cases, and explicit directions for its proper performance are here given.

It is a practical and very useful little book, and one well worth attentive perusal.

---

TRASACTIONS OF THE TWENTY-NINTH ANNUAL MEETING OF THE MEDICAL SOCIETY OF NORTH CAROLINA, AND CONJOINT SESSION OF THE NORTH CAROLINA BOARD OF HEALTH, held in Concord, May 9-11, 1882.

This paper-bound volume of 260 pages contains the proceedings, business and scientific, of the last session of the Medical Society of North Carolina and also of the Board of Health of that State. From the showing made in this report we should infer that the Society is in a healthy condition financially and otherwise. It seems, however, that no appropriation is made for the support of the Board of Health. This is radically wrong. If the board is serviceable to the State it should be well sustained, if it is worth nothing it had better be dissolved. It is unreasonable and unjust for a State ask or receive the service of a citizen and refuse to make adequate return, and medical men do themselves and their profession injury by volunteering their services under these circumstances. The State is not a proper subject for charity, and hence gratuitous service lowers the dignity of the profession and tends to lessen the respect which the public is usually willing to accord it. We trust that this feature of the law in North Carolina will soon be changed, for we are sure that this board is worthy of better things.

